# Bradycardia Related to Remdesivir During COVID-19: Persistent or Permanent?

**DOI:** 10.7759/cureus.19919

**Published:** 2021-11-26

**Authors:** Mani Maheshwari, Hemanthkumar Athiraman

**Affiliations:** 1 Hospital Medicine, Banner Health, Mesa, USA

**Keywords:** arrythymia, adult hospital medicine, covid-19, remdesivir, drug induced bradycardia

## Abstract

Remdesivir is an antiviral that inhibits RNA-dependent RNA polymerases of coronaviruses, including severe acute respiratory syndrome coronavirus 2 (SARS-CoV-2). It is a cornerstone of therapy for hospitalized patients with coronavirus disease 2019 (COVID-19), especially those worsening from a respiratory standpoint despite being started on antibiotics, dexamethasone, zinc, and vitamin C. This is a case report describing two COVID-19-positive patients with bradycardia after starting remdesivir. Once remdesivir was discontinued, one patient corrected to normal sinus rhythm, and the other continued with persistent bradycardia.

## Introduction

Remdesivir has come to light as the treatment of choice for severe coronavirus disease 2019 (COVID-19) infections since May 2020 [1]. Remdesivir is a nucleotide analog prodrug requiring activation to its triphosphorylated form, yielding a substrate for RNA-dependent RNA polymerases. Remdesivir triphosphate (RTP) incorporates into the growing RNA product by RNA-dependent RNA polymerase of coronaviruses, including severe acute respiratory syndrome coronavirus 2 (SARS-CoV-2), soon blocking RNA synthesis [2]. Previously, remdesivir was studied to treat the Ebola virus but failed in a randomized control trial done during an Ebola outbreak [3]. The most common side effects of remdesivir include an increase in hepatic enzymes, nausea, hypersensitivity, and infusion-related anaphylactic reactions [4]. This case report focuses on bradycardia during remdesivir treatment in two COVID-19-positive patients.

## Case presentation

Case 1

**Figure 1:**
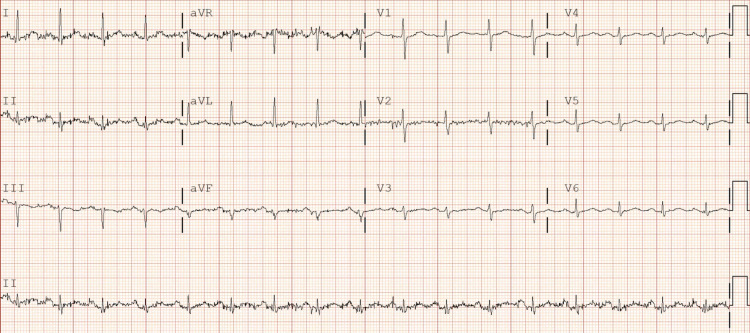
Initial EKG shows sinus tachycardia with left axis deviation; QTc 474 ms

**Figure 2:**
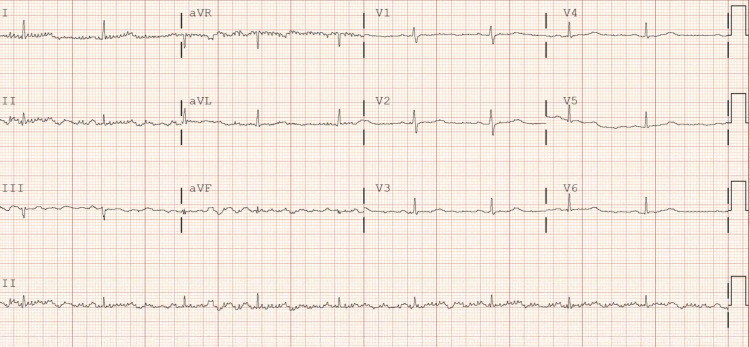
After three doses of remdesivir, EKG shows sinus bradycardia with nonspecific intraventricular conduction delay; QTc 485 ms

**Figure 3:**
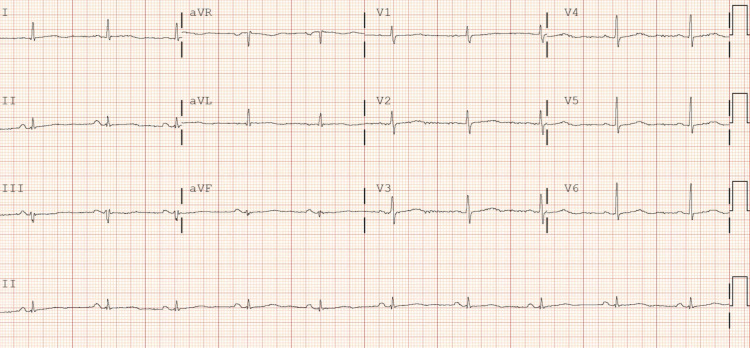
Two and a half days after the discontinuation of remdesivir, EKG shows NSR; QTc 468 ms NSR: normal sinus rhythm

A 54-year-old Caucasian female, without significant past medical history, unvaccinated for COVID-19 presented with shortness of breath, cough, myalgias, nausea, vomiting, diarrhea, and fevers a week starting with headache. Upon initial evaluation in the emergency room, vital signs were as follows: blood pressure (BP) was 115/77 mmHg, heart rate (HR) was 103 beats per minute (bpm), temperature was 99.0°F, and oxygen saturation was 84% on room air. Lab work showed nasopharyngeal swab positive for SARS-CoV-2, elevated D-dimer (772 ng/mL), elevated international normalized ratio (INR) (1.3), hyperglycemia (117 mg/dL), hyponatremia (130 mmol/L), hypokalemia (3.3 mmol/L), hypochloremia (91 mmol/L), elevated liver enzymes (aspartate aminotransferase {AST}: 157 U/L, alanine aminotransferase {ALT}: 87 U/L), elevated N-terminal pro b-type natriuretic peptide (NT-proBNP) (508 pg/mL), and elevated troponin (13 ng/L). Chest x-ray showed bilateral infiltrates. CT chest with contrast showed bilateral pneumonia. The patient was admitted to the telemetry unit and started on ceftriaxone, azithromycin, dexamethasone, and remdesivir. Initial EKG on admission showed sinus tachycardia and left axis deviation with HR of 101 bpm (Figure 1). After three days of remdesivir, EKG was repeated and showed sinus bradycardia with nonspecific intraventricular conduction delay, with HR of 57 bpm (Figure 2). Third day after discontinuing remdesivir, the patient developed a transient arrhythmia noted on telemetry which resolved within a few seconds. This prompted nurse to get an EKG which showed normal sinus rhythm (Figure 3). Potassium levels were low initially and after repletion potassium normalized on day two of hospital stay. Magnesium levels were normal throughout the hospital stay.

Case 2

**Figure 4:**
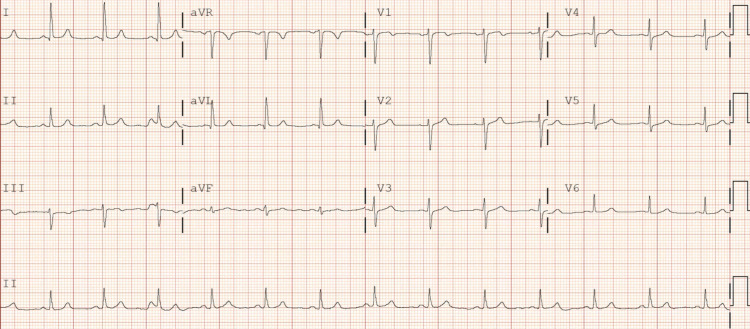
Initial EKG shows normal sinus rhythm; QTc 391 ms

**Figure 5:**
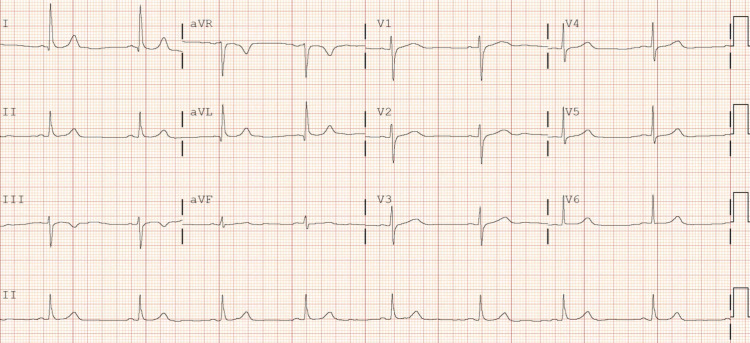
After two doses of remdesivir, EKG shows bradycardia; QTc 434 ms

A 54-year-old Hispanic female with a past medical history of type 2 diabetes mellitus, unvaccinated for COVID-19 presented with shortness of breath, cough, and pleuritic chest pain for four days. Upon initial evaluation in the emergency room, vital signs were as follows: BP was 118/63 mmHg, HR was 80 bpm, temperature was 103.1°F, and oxygen saturation was 91% on room air. Lab work showed nasopharyngeal swab positive for SARS-CoV-2, leukopenia (WBC: 3.8x10^3^/uL), elevated D-dimer (514 ng/mL), hyperglycemia (126 mg/dL), elevated liver enzymes (AST: 224 U/L, ALT: 175 U/L), elevated c-reactive protein (CRP) (129.8 mg/L), and elevated respiratory procalcitonin (0.26 ng/mL). Chest x-ray showed patchy bilateral lung opacities. CT chest with contrast showed moderate bilateral pulmonary infiltrates. The patient was admitted to the telemetry unit and started on ceftriaxone, azithromycin, and dexamethasone. EKG on admission showed normal sinus rhythm with HR of 80 bpm (Figure 4). The day following admission, the patient was started on remdesivir. After two doses of remdesivir, the patient developed severe sinus bradycardia with HR of 30-40 bpm, and remdesivir was discontinued (Figure 5). She continued to have bradycardia with HR of 45-60 bpm persistently throughout the hospitalization. Potassium and magnesium levels stayed within normal limits for this patient throughout the hospital stay.

## Discussion

A multicenter retrospective analysis showed COVID-19 patients with bradycardia had a significant increase in mortality [5]. Though this study did not involve remdesivir, multiple case reports are published showcasing remdesivir potentially causing bradycardia [6]. Unfortunately, the mechanism of remdesivir causing bradycardia is not well-established, but there are some theories found in the literature review.

One theory states that remdesivir can act on the sinus node, causing bradycardia and conduction delay because of remdesivir's structural similarity with adenosine [7-9]. Another possible mechanism, even though remdesivir is safe and studies have shown that its affinity towards viral RNA polymerase is >500 times than human mitochondrial RNA polymerase, the potential for involving human mitochondrial RNA polymerase still exists, causing mitochondrial dysfunction and cardiotoxicity [10,11]. Furthermore, an in vitro study reported time-dependent worsening cytotoxic effects of remdesivir on cardiomyocytes infected with SARS‐CoV‐2 infection measured at 24 hours and 48 hours [12]. Scientists should do more research to understand the mechanism of bradycardia induced by remdesivir.

The two COVID-19 patients presented in this case report required 40 L 100% FiO_2_ while being treated with baricitinib, antibiotics, and steroids. Cardiologists saw both patients for evaluation and treatment of bradycardia. After discontinuing remdesivir, bradycardia resolved in case 1 and persisted in case 2. The cardiologist recommended patient remain on telemetry and ordered an echocardiogram, which showed a normal ejection fraction of 55-60%. A pacemaker was considered/discussed by cardiology however was not indicated.

## Conclusions

It is imperative to note the possibility of adverse cardiovascular effects in severe COVID-19 patients undergoing treatment with remdesivir. COVID-19 patients who develop bradycardia while on remdesivir therapy especially with preexisting comorbidities should be closely monitored on telemetry. Additionally, daily lab examinations, frequent EKGs, electrolyte replacement, and avoiding medications that may prolong QTc such as azithromycin ondansetron, etc. should be considered. Furthermore, patients with persistent bradycardia upon discharge should follow up with cardiology.
